# Dynamic Response of Angle Ply Laminates with Uncertainties Using MARS, ANN-PSO, GPR and ANFIS

**DOI:** 10.3390/ma14020395

**Published:** 2021-01-14

**Authors:** Bharat Bhushan Mishra, Ajay Kumar, Jacek Zaburko, Barbara Sadowska-Buraczewska, Danuta Barnat-Hunek

**Affiliations:** 1 Department of Civil Engineering, National Institute of Technology Patna, Patna 800005, India; bbm101993@gmail.com (B.B.M.); sajaydce@gmail.com (A.K.); 2 Faculty of Environmental Engineering, Lublin University of Technology, Nadbystrzycka St. 40B, 20-618 Lublin, Poland; j.zaburko@pollub.pl; 3 Faculty of Civil Engineering and Environmental Sciences, Bialystok University of Technology, Wiejska St. 45A, 15-351 Bialystok, Poland; barbara.sadowska@pb.edu.pl; 4 Faculty of Civil Engineering and Architecture, Lublin University of Technology, Nadbystrzycka St. 40, 20-618 Lublin, Poland

**Keywords:** mode shape, angle ply laminate, finite element, Monte Carlo Simulation, MARS, GPR, ANN-PSO, ANFIS

## Abstract

In the present work, for the first time, free vibration response of angle ply laminates with uncertainties is attempted using Multivariate Adaptive Regression Spline (MARS), Artificial Neural Network-Particle Swarm Optimization (ANN-PSO), Gaussian Process Regression (GPR), and Adaptive Network Fuzzy Inference System (ANFIS). The present approach employed 2D *C*^0^ stochastic finite element (FE) model based on the Third Order Shear Deformation Theory (TSDT) in conjunction with MARS, ANN-PSO, GPR, and ANFIS. The TSDT model used eliminates the requirement of shear correction factor owing to the consideration of the actual parabolic distribution of transverse shear stress. Zero transverse shear stress at the top and bottom of the plate is enforced to compute higher-order unknowns. *C*^0^ FE model makes it commercially viable. Stochastic FE analysis done with Monte Carlo Simulation (MCS) FORTRAN inhouse code, selection of design points using a random variable framework, and soft computing with MARS, ANN-PSO, GPR, and ANFIS is implemented using MATLAB in-house code. Following the random variable frame, design points were selected from the input data generated through Monte Carlo Simulation. A total of four-mode shapes are analyzed in the present study. The comparison study was done to compare present work with results in the literature and they were found in good agreement. The stochastic parameters are Young’s elastic modulus, shear modulus, and the Poisson ratio. Lognormal distribution of properties is assumed in the present work. The current soft computation models shrink the number of trials and were found computationally efficient as the MCS-based FE modelling. The paper presents a comparison of MARS, ANN-PSO, GPR, and ANFIS algorithm performance with the stochastic FE model based on TSDT.

## 1. Introduction

In the 20th century, a comprehensive application of evolutionary computation control has led to the evolution of very efficient finite element models to analyze complex structural problems. Regardless of the developments in the computation that thoroughly aids the finite element model, the computational cost and time to perform finite element simulations for the analysis of complex engineering structure make it infeasible and uneconomical. The dynamic response of angle ply laminates with uncertainties using FE software is a complex phenomenon and requires significant computational facilities. This makes the application of finite element models quite inappropriate in Monte Carlo Simulations (MCS). MCS requires very intensive computation. A huge number of simulations are required for MCS-dependent stochastic analysis. In such instances, it will be quite fruitful to work with soft computational models, i.e., the application of artificial intelligence in complex structural engineering problems. The application of soft computation models in characterizing the probabilistic reply produced due to the uncertainty present in composite structure has significant demand for assessing the overall response of the composite structure. A substantial application of the laminated composite plates in civil, aerospace, automobile, and marine works has made it a popular research subject so that optimum performance could be achieved. The vibration produced in the plate is a function of the stacking angle, skew angle, and material properties of the laminated composite plate. Hybrid means that more than one material is used in the production of a composite structure. A hybrid angle ply laminated composite plate provides excellent mechanical behavior. The basic characteristics of a hybrid angle ply laminated composite plate are affected by the sheet-to-sheet assembly of ply and deviations in material properties of ply along with the thickness. It has a complex production and fabrication process, which led to the variation in different structural properties from its mean value. Thus, an appropriate explanation and suitable understanding of the actual response generated due to variation produced are required. For this, it is primarily necessary to account for all the inherent variations produced in the production and fabrication process. With conventional methods like finite element analysis aided with Monte Carlo Simulation (MCS), stochastic vibration analysis of hybrid composite plate is uneconomical and time-consuming; henceforth, the application of the soft computing model for assessing the response generated in the plate due to unavoidable uncertainty could lead the researcher to the new insight. Since a variety of soft computation models are available, choosing a particular model for uncertainty evaluation in the composite structure may give rise to an obvious question of why this technique is better than others and on what basis the present soft computation model is selected. This question is answered with a proper literature survey and by showing the merits of the present soft computing model over the previously used model. Further, this paper presents a broad comparative assessment of a few state-of-the-art soft computation models with each other and with the most reliable Monte Carlo Simulation-Based Finite Element Models (MCS-FEM). Various soft computation models presented in the paper are Gaussian Process Regression (GPR), Multivariate Adaptive Regression Spline (MARS), Particle Swarm Optimization Aided Artificial Neural Network (PSO-ANN), and Adaptive Network Fuzzy Inference System (ANFIS). The first two soft computing models are state-of-the-art regression models and the other two models are an advanced version of Artificial Neural Networks (ANN) with efficient learning capability. A brief literature survey on the analysis of the composite structures and different soft computation modelling techniques are presented below.

Reddy and Khdeir [1] presented buckling and vibration analysis of composite laminates under different boundary conditions. For the purpose, Reddy and Khdeir [1] used Classical Plate Theory (CPT), first-order plate theory, and third-order plate theory and concluded that CPT over-predicts the natural frequencies, but at the same time, higher-order theory gives much more accurate results than CPT. Fares and Zenkour [2] performed free vibration and buckling analysis of laminated composite plates with various plate theories and concluded that classical plate theory is inadequate in presenting an accurate response of the structure. Lin [3] studied the reliability prediction of the laminated composite plate with random system parameters subjected to a transverse load. Using *C*^0^ finite element and MCS, Singh et al. [4] presented a study over nonlinear analysis of a composite plate with material uncertainty. Kayikci and Sonmez [5] studied the design of composite laminates for the optimization of the frequency response of the composite plate. With random field properties and model uncertainty, Batou and Soize [6] presented stochastic modelling and identification of an uncertain ambiguous computational dynamic model. Mahi et al. [7] adopted the hyperbolic shear deformation theory for free vibration analysis of isotropic, FG, sandwich laminated composite plates. Using *C*^0^ finite element method based on higher-order shear deformation theory (HSDT), Kumar and Chakrabarti [8] performed a failure analysis of the laminated composite skew plate. Using nine-noded 2D *C*^0^ isoparametric element, Ansari et al. [9] studied CNT-reinforced functionally graded plates (FGP) for flexural and free vibration. Chaubey et al. [10] presented the vibration of laminated composite shells with cut-outs using *C*^0^ finite element formulation based on TSDT. Using first-order shear deformation theory (FSDT), Chaudhuri et al. [11] studied five-mode shape analysis of hyper shell with cut-out and concluded that free vibration mainly depends upon boundary conditions rather than other parameters. With improved shear deformation theory (ISDT), Anish et al. [12] analyzed bi-axial buckling of a laminated composite plate with cut-out and additional mass. By modelling uncertain material properties of FGPs with a multiple-imprecise-random-field model, Minh et al. [13] performed a hybrid uncertainty analysis of FGPs. Using a four-variable quasiHSDT, Khiloun et al. [14] presented an analytical model of bending and vibration of thick advanced composite plates. Using the smoothed particle hydrodynamics and finite element model, Zhou et al. [15] analyzed laminated composite plates for bird impact resistance. Dhakal and Sain [16] investigated the effect of unidirectional carbon fiber hybridization on the enhancement of mechanical properties of flax epoxy composite laminates. With hybrid titanium-carbon laminates subjected to low-velocity impact, Jakubczak et al. [17] investigated it for various layer thicknesses. Ostapiuk and Bieniaś [18] performed fracture analysis and shear strength of aluminium/CFRP and GFRP adhesive joint in fiber metal laminates.

GPR is a state-of-the-art regression model with a Bayesian and statistical theory framework [19]. It is a kind of probabilistic regression that is extensively used for high-dimensional problems. When compared with other regression models like ANN, SVM, Random forest method, etc., GPR is easy to implement. It is very flexible and self-adaptive and hence regulates hyperparameters very conveniently. Due to these advantages, GPR is widely used to decipher approximation problems and capture the complex relationship between the variables. Besides, GPR gives uncertainty estimates for predictions and thereby makes the regression model more relatable and efficient. Anderson et al. [20], using GPR, analyzed composite plate dynamics. Kang et al. [21], using GPR, studied the stability evaluation method for slopes and observed that GPR gives a better result than ANN and SVM. Dutta et al. [22] used GPR to predict the compressive strength of concrete and observed that the performance of GPR is better than ANN.

ANN is a powerful artificial intelligence tool used in many dynamic research areas. Many complex, diverse, and advanced applications of engineering follow the use of ANN. Though ANN captures most of the significant factors required to predict the input and output relationship, it has limitations of slow learning rate and getting trapped into local minima. To counter these limitations, the application of PSO (Particle Swarm Optimization), an optimization algorithm, has been done in the present work in conjunction with ANN. PSO is a robust global search algorithm, and it follows a commanding population-based stochastic approach to tune the weights and biases of ANN. Mahdiyar et al.’s [23] hybrid ANN-PSO model has been successfully applied in engineering. The advantage of using the PSO algorithm over the other conventional training algorithm, for instance, Back-Propagation (BP), is that the potential solution will be flown through the problem hyperspace with accelerated movements towards the best solution. Thus, in ANN-PSO, PSO during the training phase results in obtaining the weights and biases configuration, which is associated with the minimum output error. Using ANN and GA, Roseiro et al. [24] executed a study to determine the material constants of a composite laminate. Lopes et al. [25] performed a reliability analysis of a laminated composite plate using finite element analysis (FEA) and ANN using MCS as a sampling method. Tawfik et al. [26] used MCS, second-order reliability method (SORM), FEA, and ANN to implement reliability analysis of laminated composite plates in free vibration. Nguyen et al. [27] used PSO to optimize the parameters of ANN for the problem of ground response approximation in short structures and concluded that it offers higher reliability than simple ANN.

MARS is a regression model. It relates response with multiple input variables (high-dimensional data). To establish the relationship between input and response variables, MARS employs the basis functions series. To obtain good results with MARS, input variables should not be highly correlated and no data should be missing. Francis [28] in his work presented a comparison between Neural Network and MARS. For uncertainty quantification in a composite plate, Dey et al. [29] employed several algorithms along with MARS. With MARS, ELM, and ANFIS, Dutta et al. [30] predicted the strength of self-compacting concrete in compression and found that strength predicted by ANFIS is better when compared with ELM and MARS. To analyze the dynamics and stability of sandwich plates, Dey et al. [31] employed MARS using arbitrary system parameters. To design the GFRP composite, Kalnins et al. [32] employed MARS and partial polynomials.

Neuro-fuzzy schemes are widely used for those problems that have ambiguous and vague information. Artificial neural networks (ANNs) and fuzzy inference systems (FIS) are complementary machine learning technologies, and together these form adaptive intelligent systems. ANFIS is based on the Takagi–Sugeno fuzzy inference system [33]. It translates the information learned during network training into a set of fuzzy rules and represents the input/output relationship more clearly. Ceylan et al. [34] applied ANFIS and ANN to predict earthquake load reduction factors of a prefabricated industrial building and found that ANFIS is more efficient than ANN. Khademi et al. [35] applied ANN and ANFIS for predicting the strength of recycled aggregate concrete. With uniaxial in-plane compressive load subjected to steel plates with pitting corrosion, Wang et al. [36] applied ANFIS to predict the ultimate strength. Hassanzadeh et al. [37], using ANFIS and TLBO, performed an experimental and numerical investigation to estimate bridge pier scour.

These metamodels give the approximate result of the analysis very quickly, efficiently, and also provide an understanding of the relationship between the various parameters. For characterizing the probabilistic behaviour of various mode shape of the present hybrid angle ply laminated composite plate, the employed soft computation metamodel does not require reliability function in advance as in the case of the first-order reliability method (FORM) and second-order reliability method (SORM). Along with it, these metamodels provide inclusive and well-organized sample space which provides an efficient result with negligible loss in accuracy. From the last few decades, the research community has given immense attention to the stochastic analysis of complex structures, which led to more convincing analysis and design of such a complex structural system. In the present paper, the authors have adopted a layer-wise random variable approach as material properties are varied layer-wise for Monte Carlo Simulation. Monte Carlo Simulation-based stochastic approach needs a large number of simulations for characterizing the behaviour of laminated composite plate due to the random nature of stochasticity present in input parameters, and in this context, soft computation models have gained popularity as they reduce the computational load to a great extent and characterize the structure very conveniently and efficiently since metamodels require a very limited number of simulations for doing so. Most of the investigation done for the laminated composite plate is deterministic and lacks a comprehensive explanation for structural responses generated due to stochasticity in material properties. To date, a study of stochastic analysis of the mode shape of a hybrid angle ply laminated composite plate is not found in the literature with the present state-of-the-art soft computing model. The novelty of the article is a probabilistic description of four-mode shapes of hybrid angle ply laminated composite plates with MCS-FEM and efficient metamodels GPR, MARS, PSO-ANN, and ANFIS and showing the advantage of metamodels over FE model, as in earlier published articles, no or very limited work is found on mode-shape analysis using soft computation metamodels. The present work is the first attempt on mode shape analysis of a hybrid angle ply laminated composite plate using 2D *C*^0^ FE formulation based on TSDT in conjunction with MCS-FEM, GPR, MARS, PSO-ANN, and ANFIS. A comparative assessment is made for the models employed in the present work and it is shown that the ANFIS model dominates the other models in terms of precision though the result predicted by each model is in agreement with the MCS-FEM. Concerning soft computation models, mode shape analysis of laminated composite plate is not sufficient, and henceforth, comparative analysis of these techniques concerning the accuracy and computational efficiency is very rare to find in published articles.

## 2. Formulation

### 2.1. Mathematical Formulation

**Figure 1:**
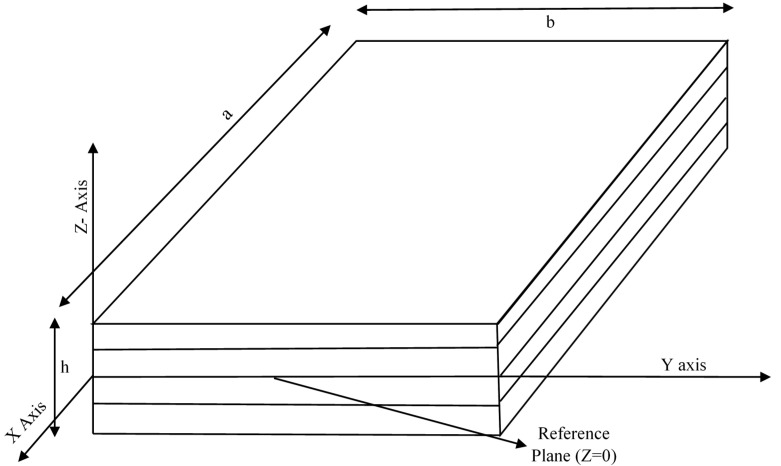
3D view of a hybrid angle ply laminated composite plate with a reference plane at Z = 0.

A hybrid angle-ply laminated composite plate with sides ‘a’ and ‘b’ along the X and Y axes is shown in Figure 1.

The reference plane of the hybrid laminated plate with random alignment and a fixed number of laminae is defined at −*z* = 0. Displacement fields as per Reddy [38] are shown in Equation (1):

(1) $$\left\{\begin{array}{c} u\\ v\\ w \end{array}\right\}=\left\{\begin{array}{c} u_{o}\\ v_{o}\\ w_{o} \end{array}\right\}+z\left\{\begin{array}{c} \theta_{x}\\ \theta_{y}\\ 0 \end{array}\right\}+z^{2}\left\{\begin{array}{c} \varphi_{x}\\ \varphi_{y}\\ 0 \end{array}\right\}+z^{3}\left\{\begin{array}{c} \chi_{x}\\ \chi_{y}\\ 0 \end{array}\right\}$$

where $u,\;u_{o}$, and $\theta_{y}$ are the displacement, midplane displacement, and rotation of normal along the X-axis respectively. $v,\;v_{o}$, and $\theta_{x}$ are the displacement, midplane displacement, and rotation of normal along the y-axis, respectively. w and $w_{o}$ are the displacements and midplane displacement along z-axis, respectively. $\varphi_{x},\;\chi_{x},\;\varphi_{y},\;\chi_{y}$ are higher-order terms. These terms are evaluated by taking the transverse shear stress zero at the top and bottom of the plate. Refined displacement fields produced by considering zero transverse shear strain at the top and bottom of laminated composite plate i.e., at ±h/2 [38], and from this the following equation is obtained:

(2) $$\begin{array}{l} u=u_{o}+z\theta_{x}\left(1-\frac{4z^{2}}{3h^{2}}\right)-\frac{4z^{3}}{3h^{2}}\left(\frac{\partial w}{\partial x}\right)=u_{o}+z\theta_{x}\left(1-\frac{4z^{2}}{3h^{2}}\right)-\frac{4z^{3}}{3h^{2}}\psi_{x}^{*}\\ v=v_{o}+z\theta_{y}\left(1-\frac{4z^{2}}{3h^{2}}\right)-\frac{4z^{3}}{3h^{2}}\left(\frac{\partial w}{\partial y}\right)=v_{o}+z\theta_{y}\left(1-\frac{4z^{2}}{3h^{2}}\right)-\frac{4z^{3}}{3h^{2}}\psi_{y}^{*}\\ w=w_{o} \end{array}$$

where $\left\{\psi_{x}^{*}\right\}=\frac{\partial w}{\partial x}$ and $\psi_{y}^{*}=\frac{\partial w}{\partial y}$, *h* is the thickness of the laminated composite plate.

In the present study, *C*^0^ finite element model has been developed based on the TSDT [38] for laminated composite plates. The actual displacement fields require *C*^1^ continuity of the transverse displacement for the finite element implementation. To avoid the difficulties associated with the *C*^1^ continuity requirement, the derivatives of *w* with respect to *x* and *y* are expressed as follows:

$\left\{\psi_{x}^{*}\right\}=\frac{\partial w}{\partial x}$ and $\psi_{y}^{*}=\frac{\partial w}{\partial y}$, which help to define all the nodal variables as *C*^0^ continuous.

Linear strain-displacement equations are written as:

(3) $$\varepsilon_{xx}=\frac{\partial u}{\partial x},\;\;\varepsilon_{yy}=\frac{\partial v}{\partial y},\;\gamma_{xy}=\frac{\partial v}{\partial x}+\frac{\partial u}{\partial y},\;\gamma_{xz}=\frac{\partial u}{\partial z}+\frac{\partial w}{\partial x},\;\;\gamma_{yz}=\frac{\partial v}{\partial z}+\frac{\partial w}{\partial y}$$

With the help of the above equation, the following equation is obtained:

(4) $$\begin{array}{l} \left\{\begin{array}{c} \varepsilon_{xx}\\ \varepsilon_{yy}\\ \gamma_{xy} \end{array}\right\}=\left\{\begin{array}{c} \varepsilon_{x0}\\ \varepsilon_{y0}\\ \varepsilon_{xy0} \end{array}\right\}+z\left\{\begin{array}{l} \theta_{x,x}\\ \theta_{y,y}\\ \theta_{x,y}+\theta_{y,x} \end{array}\right\}-\frac{4z^{3}}{3h^{2}}\left\{\begin{array}{l} \left(K_{x}+K_{x}^{*}\right)\\ \left(K_{y}+K_{y}^{*}\right)\\ \left(K_{xy}+K_{xy}^{*}\right) \end{array}\right\}\\ \left\{\begin{array}{l} \gamma_{xz}\\ \gamma_{yz} \end{array}\right\}=\left\{\begin{array}{l} \phi_{x}\\ \phi_{y} \end{array}\right\}+z\left\{\begin{array}{l} K_{xz}\\ K_{yz} \end{array}\right\}-\frac{4z^{2}}{3h^{2}}\left\{\begin{array}{l} K_{xz}^{**}\\ K_{yz}^{**} \end{array}\right\}+\frac{4z^{3}}{3h^{2}}\left\{\begin{array}{l} K_{xz}^{*}\\ K_{yz}^{*} \end{array}\right\} \end{array}$$

where $\left\{\varepsilon_{xo},\;\varepsilon_{xyo},\;\varepsilon_{yo}\right\}=\left\{u_{o,x},\;u_{o,y}+v_{o,x},\;v_{o,y}\right\}$, $\left\{\phi_{x},\;\phi_{y}\right\}=\left\{w_{o,x}+\theta_{x},\;w_{o,y}+\theta_{y}\right\}$, $\left\{K_{x},\;K_{x}^{*},\;K_{y},\;K_{y}^{*},\;K_{xy},\;K_{xy}^{*}\right\}=\left\{\theta_{x,x},\;\psi_{x,x}^{*},\;\theta_{y,y},\;\psi_{y,y}^{*},\;\theta_{x,y}+\theta_{y,x},\;\psi_{x,y}^{*}+\psi_{y,x}^{*}\right\},$ and $\left\{K_{xz}^{**},\;K_{yz}^{**}\right\}=\left\{\theta_{x}+\psi_{x}^{*},\;\theta_{y}+\psi_{y}^{*}\right\}.$

Henceforth, by adopting the standard procedure of FEM, the element matrices are assembled which results in global stiffness matrices.

### 2.2. Finite Element Formulation

**Figure 2:**
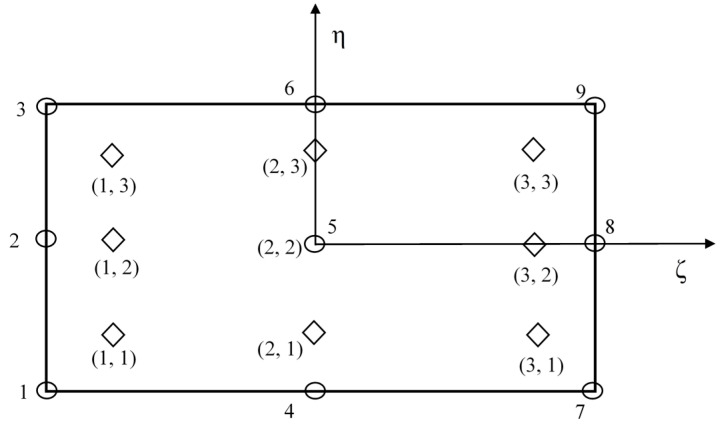
Nine-noded isoparametric elements with typical node numbering and Gauss points.

Figure 2 shows a nine-noded isoparametric *C*^0^ element. Each node has seven unknowns. It is utilized to frame the FE model employed in the present study.

**Figure 3:**
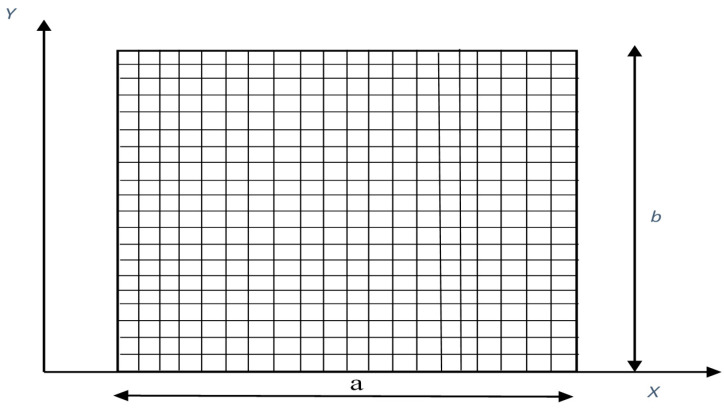
Hybrid angle ply laminated composite plate with 20 × 20 meshing.

Figure 3 shows the present plate with a mesh size of 20 × 20.

For a typical element on the middle surface, unknown nodal vector {d} is expressed by:

(5) $$\left\{d\right\}=\overset{9}{\underset{i=1}{\sum }}N_{i}\left(x,y\right)\left\{d_{i}\right\}$$

here, $N_{i}$ is the interpolating shape function and $d_{i}$ is an unknown nodal vector that belongs to the *i*^th^ node.

Concerning the global displacement, the generalized mid-plane strains can be expressed as:

(6) $$\left\{\bar{\varepsilon }\right\}=\overset{9}{\underset{i=1}{\sum }}\left[B_{i}\right]\left\{d_{i}\right\}$$

here, $\left[B_{i}\right]$ is the interpolation function differential operator matrix.

Using the expression mentioned above, the element stiffness matrix is evaluated and stated as:

(7) $$\left[K_{e}\right]=\int_{-1}^{1}\int_{-1}^{1}{\left[B\right]}^{T}\left[D\right]\left[B\right]\left|j\right|dxdy$$

(8) $$\left[M_{e}\right]=\int_{-1}^{1}\int_{-1}^{1}{\left[N\right]}^{T}\left[m\right]\left[N\right]\left|j\right|dxdy$$

here, [*N*], [*m*], and |j| represent shape function, inertia matrix, and determinant of the Jacobian matrix, respectively.

The natural frequency produced during free vibration is evaluated using the following equation:

(9) $$\left[K\right]-w_{n}^{2}\left[M\right]=\left[0\right]$$

where *w* is the fundamental natural frequency of the hybrid angle ply laminated composite plate. The matrices *K* and *M* are the element stiffness matrix.

### 2.3. Gaussian Process Regression (GPR)

Gaussian process (GP) is a normal stochastic process, i.e., it has a joint Gaussian distribution [39]. Gaussian process (*f*(*x*)) is parametric, and it is parameterized with mean and kernel or covariance function evaluated at points *x* and *x*′. A Gaussian function is defined in the equation below:

(10) $$\begin{array}{l} m\left(x\right)=E\left(f\left(x\right)\right)\\ Cov\left(f\left(x\right),f\left(x^{\prime }\right)\right)=k\left(x,x^{\prime }:\theta \right)=E\left(\left(f\left(x\right)-m\left(x\right)\right)\left(f\left(x^{\prime }\right)-m\left(x^{\prime }\right)\right)\right) \end{array}$$

where *θ* is hyperparameters set. Henceforth, a Gaussian process *f*(*x*) is written as:

(11) $$f\left(x\right)\sim GP\left(m\left(x\right),\;k\left(x,x^{\prime }\right)\right)$$

This implies that *f*(*x*) is distributed as a GP with mean *m*(*x*) and covariance *k* (*x*, *x*′).

GPR is a non-parametric Bayesian model which is generally used to solve non-linear regression problem. Response variable *y* can be related to input variable *x* via fundamental regression function *f*(*x*) with random uniformly distributed noise (*ε*) by the following equation:

(12) $$y_{i}=f\left(x_{i}\right)+\varepsilon$$

*ε* (noise) has mean = 0 i.e., *m*(*x*) = 0 and variance = 0 i.e., *σ_n_*^2^ = 0, and it is expressed as:

$$\varepsilon =N\left(0,\sigma_{n}^{2}\right)$$

The Gaussian process represented in the above equation becomes:

(13) $$f\left(x\right)\sim GP\left(m\left(x\right),k\left(x,x^{\prime }\right)+\sigma_{n}^{2}I\right)$$

here *I* is the identity matrix. Depending on the noise *ε* and the GP’s marginalization property, the joint distribution of the train output *y* at train points *X* and test output f* at test points X* is expressed as:

(14) $$\left[\begin{array}{c} y\\ f_{*} \end{array}\right]\sim \left(\left[\begin{array}{c} m\left(X\right)\\ m(X_{*}) \end{array}\right],\left[\begin{array}{cc} k\left(X,X+\sigma_{n}^{2}\right) & k\left(X,X_{*}\right)\\ k\left(X_{*},X\right) & k\left(X_{*},X_{*}\right) \end{array}\right]\right)$$

A kernel or covariance function is the principal element in a GPR model. Hence, choosing the appropriate kernel function is important for the evaluation of the sample function being moulded. There are several kernel functions used in literature as per the suitability of data. For the present paper, the rational quadratic covariance function is used.

### 2.4. Multivariate Adaptive Regression Spline (MARS)

MARS model is an extended version of a linear model having non-parametric nature. It is suitable for data that are linear but also contain some sort of non-linearity.

**Figure 4:**
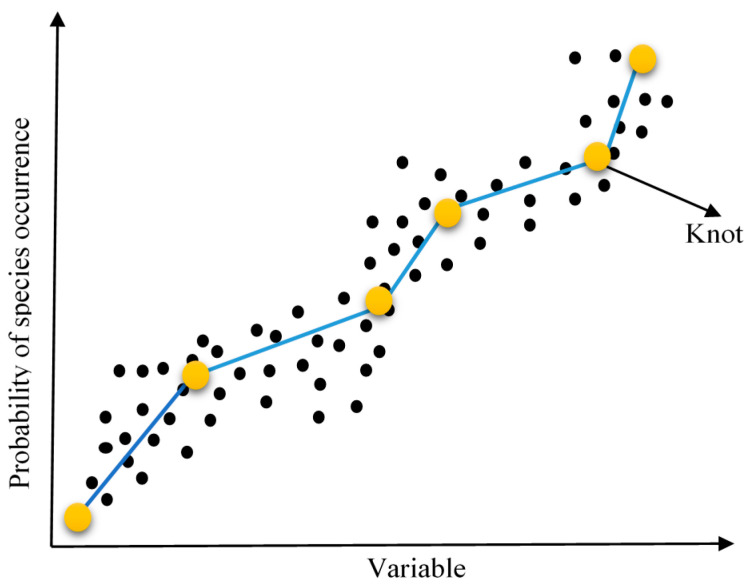
MARS regression plot with basis function and knots.

The MARS develops the model in two phases. Initially, it splits the data into splines and creates knots at end of the splines [40] as shown in Figure 4.

Basis functions characterize the data in each spline. A basis function represents a regression equation between two knots having a specific slope (Figure 4) and describes the relationship between multiple input variables and responses. MARS examines the data very efficiently to search the best spots to place the knots. The model parameters and basis function together predict the output without any prior information of associations between input and output variables. They predict the result via the relation that is learned with data divided into splines. MARS is represented by the following equation:

(15) y=g(x)+ε

where *y* is predicted response, ε is estimated error, and g(x) is a linear combination of basis function and coefficient expressed as:

(16) $$g\left(x\right)=\beta_{o}+\overset{N}{\underset{n=1}{\sum }}\beta_{n}BF\left(x\right)$$

here, *N* is the number of basis functions, $\beta_{o}$ is intercept, and $\beta_{n}$ are the coefficients corresponding to the given specific basis function (*BF*(*x*)), which is estimated by the least squares method.

### 2.5. Particle Swarm Optimization (PSO)

The PSO is an evolutionary algorithm proposed by Kennedy and Eberhart in 1997 [41]. It simulates the behavior of fish flocks. It has a fast convergence rate, high efficiency, and continuous nature [42] but lacks adaptive learning ability. Hasanipanah et al. [43] employed PSO for their work. In PSO, a fitness function that needs to be optimized is primarily defined. Afterwards, swarms are produced and spread in the high-dimensional problem space. The fitness function is to be calculated for the respective particle that holds the variables of the problem. Ultimately, the position and the velocity of the respective particle is updated as per Equations (17) and (18) until the algorithm converges [44]:

(17) $$U_{i}{}^{k+1}=wU_{i}^{k}+\alpha_{1}\cdot \left(P_{best,i}^{k}-x_{i}^{k}\right)/\Delta t+\alpha_{2}\cdot \left(G_{best,i}^{k}-x_{i}^{k}\right)$$

(18) $$x_{i}^{k+1}=x_{i}^{k}+U_{i}^{k+1}$$

*i* is the iteration number and *k* is a particle. $x_{i}$ and $U_{i}$ are position vector and velocity vectors of iteration *i* and the instance number *k*. The vectors $P_{best,i}^{k}$ and $G_{best,i}^{k}$ are the best current local best fitness function and global fitness function. *α*_1_ = *C*_1_*r*_1_ and *α*_2_ = *C*_2_*r*_2_, where *α*_i_ represents the coefficient factor. *C*_1_ and *C_2_* are learning parameters that represent the degrees of local search and global search level, respectively. *r*_1_ and *r*_2_ represent random numbers distributed uniformly between 0 and 1. *w* is the initial weight that stores previous particle velocity during the optimization problem.

### 2.6. ANN-PSO

ANN is inspired by biological treatment mode, having the capability of learning patterns and predicting results for a problem consisting of high-dimensional space (a large number of input parameters). It maps input sets to the output sets under the environment of noisy and complex data and solves a complex practical problem. Backpropagation neural network algorithms are commonly used to train a neural network [45] and have robust adaptive learning and non-linear simulation capabilities, but they very easily fall into a local minimum. The proper design of the network is subjected to the experience of the designer and sample data with no speculative guidance. Henceforth, getting the global optimal solution with only a backpropagation neural network is unlikely. Thus, an ANN leads to a minimization problem and easily falls into the local minimum. The mathematical formulation for ANN can be written as:

(19) $$y_{i}=f\left(\overset{n}{\underset{i=1}{\sum }}w_{ij}x_{i}+b_{j}\right)$$

where *x_i_* and *y_j_* are nodal values in the preceding layer *i* and present layer *j*. *n* is the total number of the nodal values received from the preceding layer. *w_ij_* and *b_j_* are weight and biases of the network and *f* is the activation function.

PSO in ANN-PSO checks the inaccuracies of the ANN by assessing the optimal values of weights and biases [27]. Henceforth in ANN-PSO weights and biases are variables, and the probable space of the problem is subjected to the interval at which weight and biases vary. In terms of RMSE, the fitness function of *i*^th^ particle is defined as [46]:

(20) $$E(w_{i},b){\sqrt{\frac{1}{s}\overset{s}{\underset{k=1}{\sum }}\left[\overset{o}{\underset{l=1}{\sum }}\left(T_{kl}-P_{kl}\left(w_{i},b_{i}\right)\right)\right]}}_{i}$$

where *E*, *T_kl_*, and *P_kl_* represent fitness value, target value, and predicted value, respectively. *w_i_* are weights, *b_i_* represents biases, *S* is training samples, and O is neurons.

Following steps is followed for building the PSO-ANN model:

1. A neural network is created with initial weights and biases by fixing the number of neurons in the hidden layer.
2. Weights and biases are updated regularly to represent the particle location in the high-dimensional (*N*) space of the problem.
3. For each particle in a particular iteration, output values can be predicted, and correspondingly, the value of cost function in Equation (20) is evaluated.
4. For particular populations and iterations, PSO updates the location of particles until the fitness function is not minimized.

### 2.7. Adaptive Network Fuzzy Inference System (ANFIS)

ANFIS is a combination of the Fuzzy Inference System (FIS) and Artificial Neural Network (ANN) for solving complex and nonlinear problems. In the present work, the Takagi-Sugeno system is employed as FIS. To simplify the model used, it is assumed that the framework of ANFIS comprises two inputs (*x, y*) and one output (*F*). Thus, a fuzzy rule based on the Takagi–Sugeno type can be represented as below [47].

Rule 1:

If *x* is *A*_1_ and *y* is *B*_1_, then

(21) $$F_{1}=a_{1}x+b_{1}y+r_{1}$$

Rule 2:

If *x* is *A*_2_ and y is *B*_2_, then

(22) $$F_{2}=a_{2}x+b_{2}y+r_{2}$$

where *A*_1_, *A*_2_, *B*_1_, and *B*_2_ are nonlinear parameters and membership functions for inputs, and *a*_1_*, a*_2_, *b*_1_, *b*_2_, *r*_1_, and *r*_2_ are linear and output’s function (*F*) parameters. The ANFIS architecture includes five different layers termed as a fuzzy layer, product layer, normalized layer, de-fuzzy layer, and output layer. Each layer has unique functions. The function of each layer is depicted in Equations (23)–(28).

In the first layer, the membership relationship including the input and output functions can be written as:

(23) $$F_{i1}=\mu A_{i}\left(x\right);i=1,2,3.$$

(24) $$F_{i1}=\mu B_{i}\left(y\right);i=1,2,3\ldots$$

where *F*_*i*__1_ and *F*_*i*__1_ indicate the output functions, and *μA*_*i*_(*x*) and *μB*_*i*_(*y*) show membership functions.

In the second layer, each node computes the ‘firing strength’ (*w*_*i*_) of each rule. The output (*F*_*i*__2_) of this layer is the product of input signals and is represented as:

(25) $$F_{i2}=w_{i}=\mu A_{i}\left(x\right)\times \mu B_{i}\left(y\right),\;\;\;\;i=1,2,3.$$

Layer 3 gives normalized firing strength (*F*_*i*__3_), or it can be said that the weight function is under normalization as:

(26) $$F_{i3}=w=w_{i}w_{1}+w_{2},\;\;\;\;i=1,2,3.$$

In the fourth layer, the output of the preceding layer is multiplied with Equation (23) and Equation (24), and the output (*F*_*i*__4_) of the 4th layer is stated as:

(27) $$F_{i4}=w_{i}f_{i}=w_{i}\left(a_{i}x+b_{i}y+r_{i}\right),\;\;\;\;i=1,2,3,\ldots$$

The fifth layer calculates overall output as follows, where $F_{i5}$ represent response predicted by the ANFIS model:

(28) $$F_{i5}=\mathrm{overall}\;\mathrm{output}=\sum^{\;}w_{i}f_{i}=\sum^{\;}w_{i}f_{i}\sum^{\;}w_{iiii}$$

In the ANFIS structure, the first layer and the fourth layer include parameters that can be changed over time. The first layer contains the nonlinearities of the precursor parameters, while the fourth layer contains the linear result parameters. Both of these parameters can be modified and updated with a learning method that trains both of these parameters and also adapts to their conditions.

## 3. Model Development

### 3.1. Data Preparation

Following the random variable framework, design points are selected from the dataset generated using MCS based FEM. With these design points, the frequency of hybrid angle ply laminated composite plate is calculated using the deterministic finite element model (FEM) coded in FORTRAN. Design points and corresponding frequency evaluated by FEM are used for training the metamodels. The trained metamodels are further used to predict the frequency of the hybrid angle ply laminated composite plate.

### 3.2. GPR Model Architecture

The parameters for the mean and covariance (kernel) function are called hyperparameters. These hyperparameters describe the performance of the GPR model. Hyperparameters allied with the mean and kernel functions must be learned to properly train and formulate the Gaussian process regression model, and it is achieved with optimization or sampling techniques. However, the extensively used approach is to maximize the log marginal likelihood. shown as:

(29) $$log\;p(y|X,\theta )=-\frac{1}{2}y^{T}{\left(K+\sigma_{n}^{2}I\right)}^{-1}y-\frac{1}{2}log|K+\sigma_{n}^{2}I|-\frac{n}{2}log\;2\pi$$

where *y^T^* represents the transpose of vector *y* and *θ* represents a vector containing entire hyperparameters.

In the proposed GPR model, a simple mean function with constant *c* is used. Various other hyperparameters used in the proposed GPR model are rational quadratic covariance function as covariance (kernel) function, the likelihood function is likGauss, and the inference method used is infGaussLik. The present inference method is an exact inference method used only when the likelihood function likGauss is used. The equation proposed for rational quadratic covariance function is mentioned below:

(30) $$k\left(x_{i},x_{j}\right)=\sigma_{f}^{2}exp{\left[1+\frac{d^{2}}{2\alpha \ell^{2}}\right]}^{-\alpha }$$

### 3.3. MARS Model Architecture

MARS is proposed as a flexible regression model for high-dimensional data. It uses a piecewise linear regression basis function and establishes the relationship between input and output value. The parameter associated with each basis function is spontaneously evaluated by the data from side to side via a forward/backward iterative approach.

MARS builds the model in two stages: first, it performs forward selection, and then the backward deletion stage is performed. In the first stage, MARS only assumes the intercept factor and then subsequently in each iteration adds redirected sets of basis functions such that least training error is produced.

After the first stage of modeling, the formulated model over-fits the data, and hence the backward deletion stage is employed. In one phase at a time, MARS removes one insignificant basis function such that the end model has only the intercept factor. Finally, when the backward phase is over, the model with the least value of Generalized Cross-Validation (GCV) is nominated as the final model. GCV estimates predicted Mean Squared Error for the MARS, and it is evaluated as [40]:

(31) $$GCV=\frac{MSE\left(train\right)}{{\left(1-\frac{p_{eff}}{n}\right)}^{2}}$$

where *MSE_train_* represents the mean square error of the model for the training data set, *n* is the number of training data, and *p_eff_* is the number of effective parameters.

### 3.4. ANN-PSO Model Architecture

After performing several trials at a different number of neurons, for the present study, it is found that the model performs at its best with five numbers of neurons. With PSO, the various parameters that need to be optimized are *C*_1_, *C*_2_, *w*, and population (swarm) size, i.e., the number of particles. The value of *C*_1_ and *C*_2_ ranges between 0 and 4. Model is simulated several times with different values of *C*_1_ and *C*_2_, and it is found that for a value of *C*_1_ = 2 and *C*_2_ = 2, the ANN-PSO model converges very quickly and gives the best fitness value. The initial value of *w* is taken as 0.1. For good results swarm size generally varies in the range of 20 to 40. To determine the optimum swarm size (population), the model is simulated several times for different swarm sizes, it is found that with a swarm size of 35, the model performs at its best. Henceforth in the present work, a swarm size of 35 is taken.

### 3.5. ANFIS Model Architecture

To obtain sufficient prediction capability using ANFIS, it must be provided with an adequate number of clusters for the model. The present ANFIS model uses fuzzy c-means clustering to formulate a fuzzy inference system, and it is subjected to the number of data being applied for training. To find the number of rules and membership function, the rule extraction method is applied. The rule extraction method mainly uses the FCM clustering function (also known as Fuzzy C-means or genfis3). The Fuzzy c-means (FCM) clustering techniques were also used to optimize the result by evaluating the set of rules that model the data and generates an initial FIS for ANFIS training. This parameter of the ANFIS model provides specifications about the number of clusters used to model the data. Details of the ANFIS parameter are mentioned in Table 1.

### 3.6. Random Input Representation

In the present work, layer-wise random input parameters are considered. Only the material property is varied, keeping other variables such as geometric property and stacking constant at a given time. The material property to which variations are provided is Young’s Modulus (*E*_1_, *E*_2_), Poisson’s ratio (*υ*_1_, *υ*_2_), and shear modulus of rigidity (*G*_12_, *G*_13_, and *G*_23_). Density remains constant in the analysis. Layerwise stochasticity in material property is written as *S*(ϖ).

(32) $$S\left(\varpi \right)=\left\{E1_{\left(1,2\right)},E2_{\left(1,2\right)},\upsilon 1_{\left(1,2\right)},\upsilon 2_{\left(1,2\right)},G12_{\left(1,2\right)},G13_{\left(1,2\right)},G23_{\left(1,2\right)}\right\}$$

In this study, hybrid angle ply is considered, which implies that laminated composite plates are made of two different materials. Angle ply means that the stacking angle would vary within range from 15° to 75°. Subscript 1 and 2 refer to two different materials that are used in the present hybrid angle ply laminated composite plate. Variations in material 1 and material 2 are taken as ±10, ±10, ±10, ±10, ±10, ±10, and ±10, respectively, and are in agreement with industry standard. A total of 14 parameters are varied in the present analysis, seven from each material.

## 4. Results and Discussion

The FE model used in the present work is based on TSDT and has been used in the analysis of the fundamental natural frequency of hybrid angle ply laminated composite plate. The computer code of the above finite element formulation is done in FORTRAN 90. In Table 2a, first, the convergence study of the developed finite element code is done to check the stability of the solution at a suitable mesh size. Further, the comparison of the present results with journal papers by Mandal et al. [48] and Reddy and Chao [49] has been done. Mandal et al.’s [48] and Reddy and Chao’s [49] work is based on first-order shear deformation theory (which considers linear transverse shear stress variation across the thickness of the plate) using finite element and closed-form solutions, respectively. The present results are based on third-order shear deformation theory (which considers the realistic parabolic transverse shear stress variation across the thickness of the plate) using finite element solutions; hence, slight variation is observed with reference papers by Mandal et al. [48] and Reddy and Chao [49].

The boundary condition used in the present study is:

Simply supported (SSSS):

(33) $$\begin{array}{l} v=w=\theta_{y}=\psi_{y}^{*},\;\mathrm{at}\;x=0,a\\ u=w=\theta_{x}=\psi_{x}^{*},\;\mathrm{at}\;x=0,b \end{array}$$

The present work results are in good agreement with published results of Mandal et al. [48] and Reddy and Chao [49].

Following the deterministic approach, validation of the FE model developed for the present analysis is done. Few fundamental natural frequencies for simply supported boundary conditions at all four edges (SSSS) for two different aspect ratios 0.01 and 0.1 are evaluated and tabulated in Table 2a.

In Table 2b, the free vibration response of a simply supported cross-ply (0°/90°) square laminate obtained from present *C*^0^ finite element model has been compared with the results of Serdoun and Hamza [50] based on HSDT C^1^ finite element model.

In Table 2c, the free vibration response of a simply supported cross-ply (0°/90°) square laminate obtained from present *C*^0^ finite element model has been compared with the results of Ganapathi et al. [51] based on HSDT *C*^0^ finite element model.

It may be observed in Table 2b,c that present results are in good agreement with published works in the literature.

For the study, non-dimensional frequency is considered. The equation employed for calculation of non-dimensional frequency is given as:

(34) $$\lambda =\frac{wa^{2}}{h}\sqrt{\frac{\rho }{E_{2}}}$$

where *λ* is non-dimensional frequency, *w* is frequency obtained by FE model, *a* is the plate lateral dimension, *h* represents the thickness of the plate, *ρ* is density, and *E*_2_ is Young’s modulus in the transverse direction.

The relative dimension of the plates studied in the present work is *a* = 1, *b* = 1, and the thickness (*h*) is *a*/100. Asymmetric laminated composite plate with four different layers of the lamina are analyzed in the present work with simply supported boundary (SSSS) conditions at all four edges. For the present plate, stacking sequences for all four laminae are 15°, 30°, 45°, 60° from the top layer to the bottom layer. Figure 1 represents the present plate model. Figure 2 shows a nine-noded isoparametric plate element that is employed to analyze the present plate model.

Relative material property and covariance incorporated agreeing with the industry standard for the present study are tabulated in Table 3.

**Figure 5:**
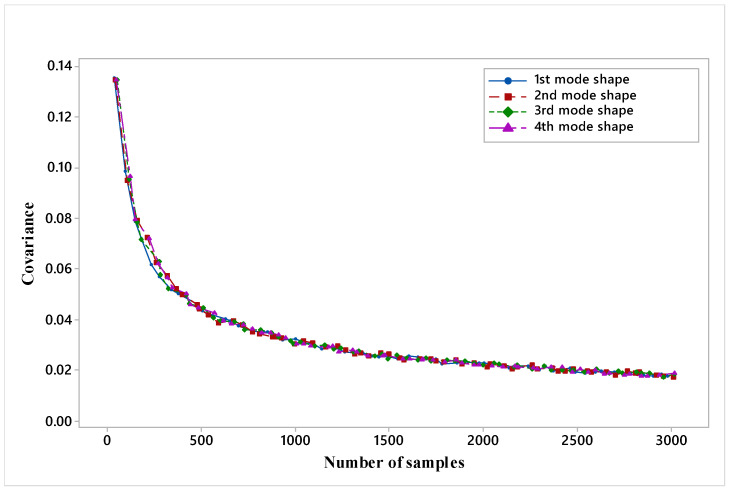
Variation of covariance with a number of iterations for hybrid angle ply laminated composite plate.

Figure 5 shows that for all four mode shapes, at an iteration equal to 3000, error becomes constant and concludes that 3000 MCS-FEM simulations are sufficient for describing the stochasticity in all four mode shapes of the present plate. Evaluation of 3000 MCS-FEM simulations is a very tedious task because it requires too much time (more than 24 h) to simulate on the workstation, which becomes a major drawback of finite element analysis. To sort out this limitation, soft computation metamodels are favored.

**Figure 6:**
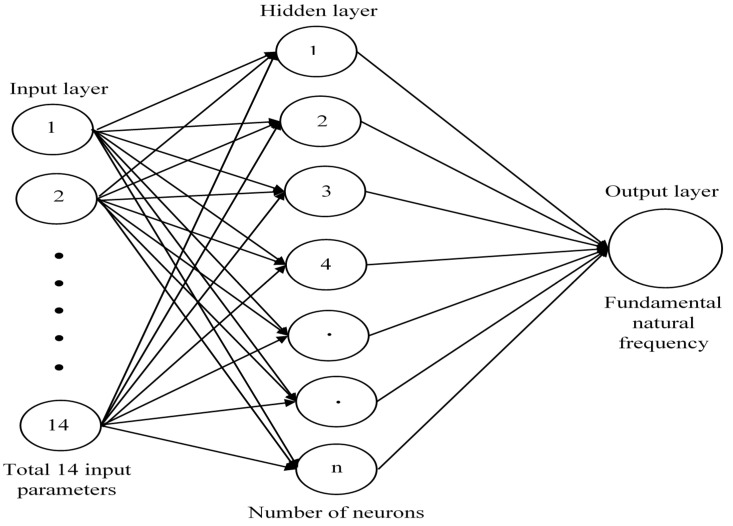
Artificial neural network (ANN) architecture.

Figure 6 shows the ANN architecture employed in the present PSO-ANN metamodel.

**Figure 7:**
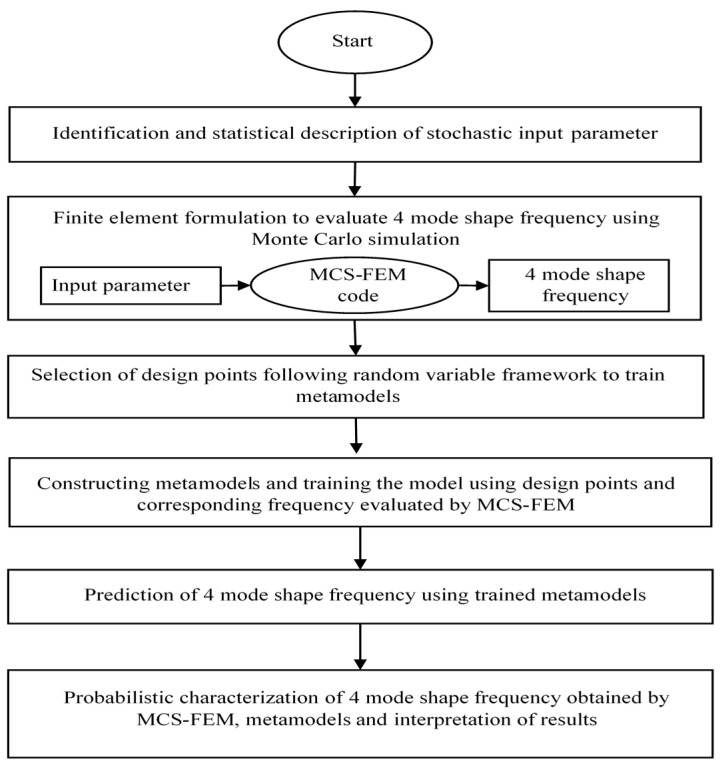
Flowchart of stochastic analysis using Monte-Carlo simulation based Finite element modeling (MCS-FEM) and metamodels.

The flow chart of the present study is shown in Figure 7.

In the present study, stochasticity in the material property as described in random input generation is provided. The relative combined effect of the input parameters includes Young’s modulus of elasticity, longitudinal shear modulus, and Poisson ratio.

**Figure 8:**
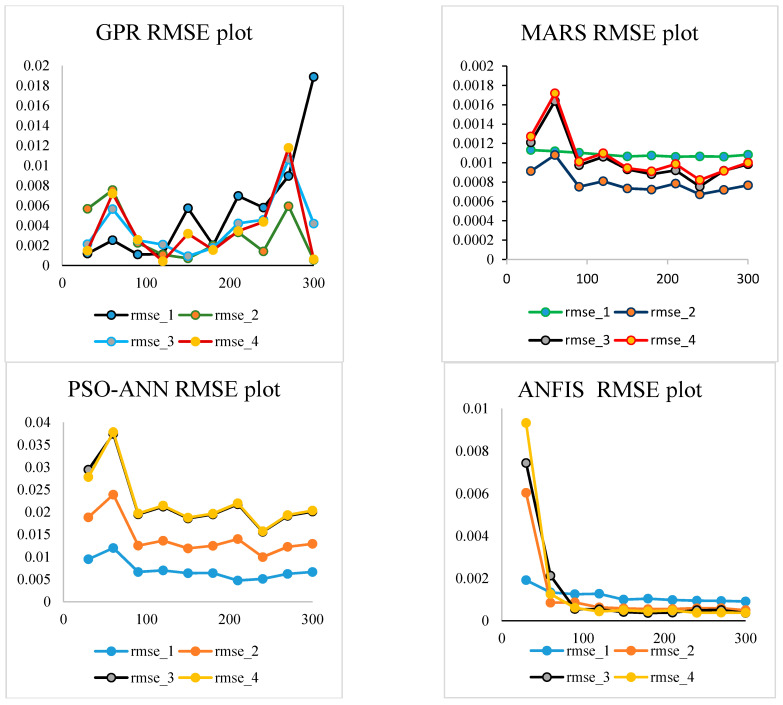
RMSE plot for predicted output by 4 metamodels for all 4 mode shape at a different number of design points from 30 to 300 (Y-axis represents RMSE value, the X-axis represents the number of design points).

Different metamodels employed in the present work are GPR, MARS, PSO-ANN, and ANFIS. These metamodels are applied to explore the predictive and representative models that could be used in place of the finite element model. Corresponding to the stochastic input variables and frequency evaluated by MCS-FEM, four mode shape frequencies are predicted by the trained metamodels. For training the metamodels, design points are selected using a random variable framework. Design points from 30 to 300 are randomly selected to train the model, and then new results are predicted. Further corresponding to each mode shape predicted frequency, root mean square error (RMSE) value is evaluated to find out the appropriate number of design points which will be sufficient to train the metamodels. Figure 8 presents the RMSE plot for various metamodels at a different number of design points for all four mode shapes.

That number of design points for which RMSE value is minimum for all four mode shapes is further used for training the metamodels from which new results are predicted. For training the GPR model, the number of design points used corresponding to the 1st–4th mode shapes are 120, 300, 150, and 300 respectively. For the MARS model, design points corresponding to the 1st–4th mode shapes are 210, 240, 240, and 240 respectively. For the PSO-ANN model, design points corresponding to the 1st–4th mode shapes are 210, 240, 240, and 240 respectively. Similarly for the ANFIS model, design points used for training corresponding to the 1st–4th mode shapes are 300, 300, 300, and 300 respectively.

For validation of these metamodels as a surrogate of the actual FE model, various scatter plots, a probability distribution plot, and statistical parameters tabulated in Table 4, Table 5, Table 6, Table 7, Table 8, Table 9, Table 10 and Table 11 are presented.

The greater the deviation of points from the diagonal line, the poorer the model appears. Less deviancy represents a more accurate model, and it validates the use of a developed surrogate model.

**Figure 9:**
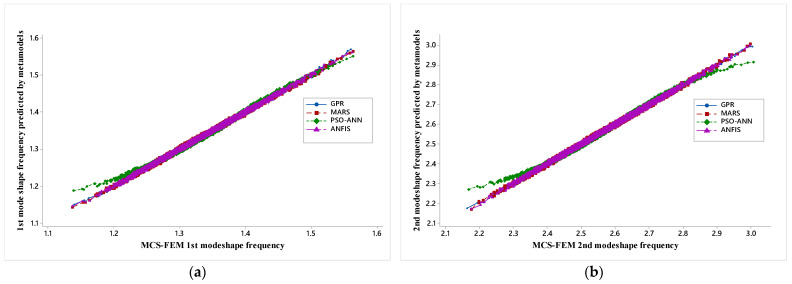

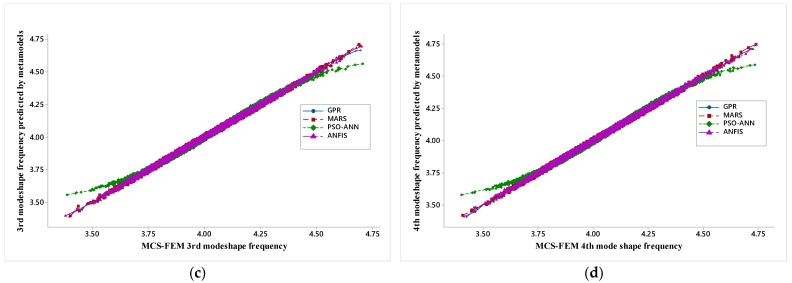
(**a**) Regression plot for 1st mode shape, (**b**) regression plot for 2nd mode shape, (**c**) regression plot for 3rd mode shape, and (**d**) regression plot for 4th mode shape.

The study is performed with stochastic variation in material property and with four mode shapes to observe the effect of stochasticity on various mode shape frequency of hybrid angle ply laminated composite plate. Figure 9a–d shows the regression plot between MCS-FEM mode shape frequency and mode shape frequency predicted by the metamodels.

For the present work, initially, four-mode shapes are analyzed with MCS-FEM, and then corresponding mode shape frequency is predicted by trained metamodels. Each figure represents a particular mode shape regression plot for four different metamodels. The regression plot of the PSO-ANN model is much staggered when compared with the GPR, MARS, and ANFIS model because PSO only tunes the parameter of ANN but it does not add inference capability to ANN as in ANFIS; FIS (fuzzy inference system) provides a reasoning capability that enhances the prediction capacity of ANN. Further regression plots for GPR, MARS, and ANFIS models nearly overlap each other and are almost aligned along the diagonal line, which shows that these metamodels are better than PSO-ANN and that results predicted by these are similar to MCS-FEM. Here, it should be noted that PSO-ANN and ANFIS are modified forms of neural network and that these models learn from the input provided and further predict the results, whereas GPR and MARS are state-of-the-art regression algorithms.

**Figure 10:**
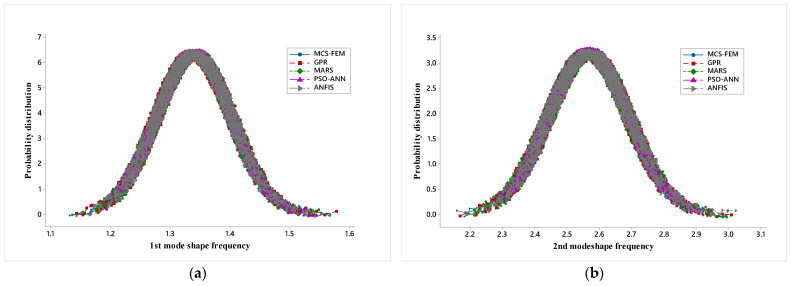

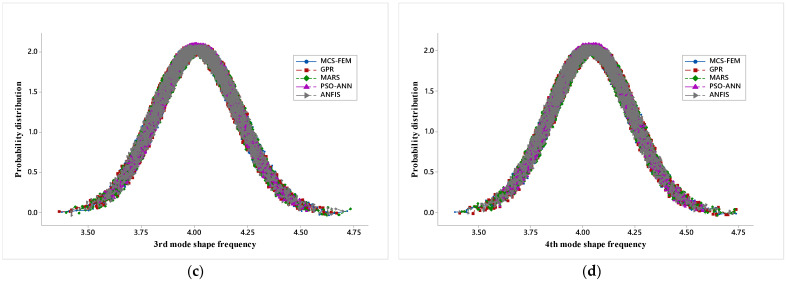
(**a**) Probability distribution (PD) plot for 1st mode shape, (**b**) PD plot for 2nd mode shape, (**c**) PD plot for 3rd mode shape and (**d**) PD plot for 4th mode shape.

Figure 10a–d represent the probability distribution plots for each mode shape and all five different models employed in the present study i.e., MCS-FEM, GPR, MARS, PSO-ANN, and ANFIS.

MCS-FEM probability distribution plot is a benchmark plot, and from this, probability distribution plots obtained from other models are compared. The probability distribution plots based on the five models nearly overlap, but it can be observed that tail endpoints of the plot lack the frequency predicted by the PSO-ANN model, whereas the frequency predicted by the rest of the model uniformly overlaps the MCS-FEM probability distribution plot. This shows that GPR, MARS, and ANFIS performances are much better than the PSO-ANN, and ANFIS is the most accurate.

**Figure 11:**
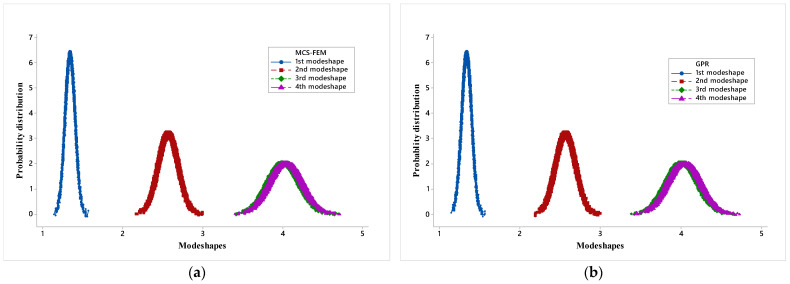

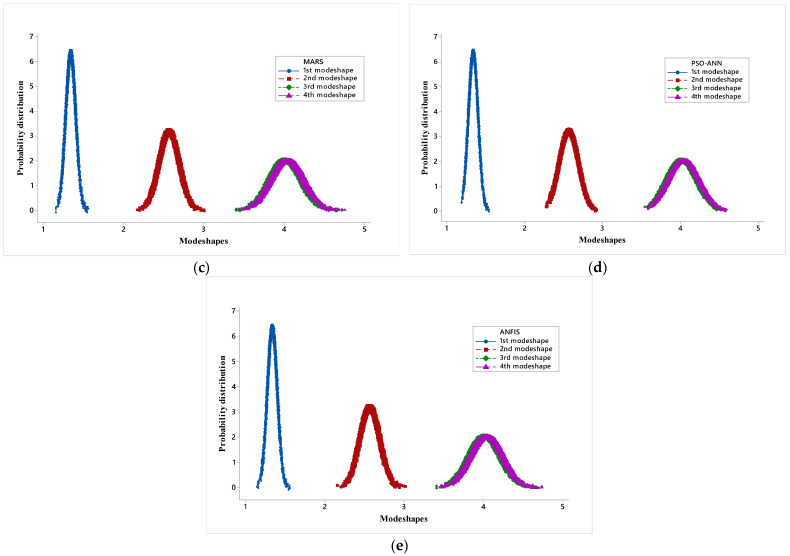
(**a**) Probability distribution (PD) plots of MCS-FEM for all four mode shapes; (**b**) (PD) plots of GPR for all four mode shapes; (**c**) (PD) plots of MARS for all 4 mode shapes; (**d**) (PD) plots of PSO-ANN for all 4 mode shapes; and (**e**) (PD) plots for ANFIS for all 4 mode shapes.

Probability distribution plots for four mode shapes frequencies of a simply supported hybrid angle ply laminated composite plate due to combined variation in the material property (*S*(ϖ)) are presented in Figure 11a–e.

As the mode shape increases, a trend is noticed that the mean and response bound increases. Here again, it can be observed that the probability distribution plot of the mode shape frequency obtained by the PSO-ANN model is somewhat different from MCS-FEM as at tail points it does not coincide with MCS-FEM. Whereas for the rest of the metamodels, the plots are nearly identical to the MCS-FEM plot. This again validates the use of metamodels in stochastic analysis of a hybrid angle ply laminated composite plate and shows that of the four models, ANFIS is most accurate in predicting the results and PSO-ANN is least accurate.

Statistics of four-mode shape frequencies obtained by MCS-FEM and frequency predicted by metamodels are summarized in Table 4, Table 5, Table 6 and Table 7. It can be observed that the result predicted by the metamodels agrees with MCS-FEM. Parameters like maximum value (Max), minimum value (Min), mean value (Mean), and standard deviation (SD) of GPR, MARS, and ANFIS are nearly identical to MCS-FEM, whereas PSO-ANN deviates from the MCS-FEM value. Of all four metamodels, the best result is obtained with the ANFIS model, and the result predicted by PSO-ANN is close to MCS-FEM, but its precision, when compared with the rest of the three models, is less. These tables further validate the use of metamodels for stochastic analysis of the mode shapes of a hybrid angle ply laminated composite plate.

Table 8, Table 9, Table 10 and Table 11 presents various statistical parameters of each mode shape that shows the precision level of various soft computation metamodels. Different statistical parameters calculated are root mean square error (RMSE), root mean square error to observations standard deviation ratio (RSR), Nash Sutcliff coefficient (NS), variance account factor (VAF), maximum determination coefficient value (*R*^2^), performance index (PI), and adjusted determination coefficient value (Adjusted *R*^2^). Optimum values of the statistical parameter presented in Table 8, Table 9, Table 10 and Table 11 are as follows: RMSE should be close to 0, NS value should be close to 1, RSR should be close to 0, VAF should be close to 100, *R*^2^ value should be close to 1, R should be close to 1, adjusted *R*^2^ should be close to 1, and PI should be greater than 1. From Table 8, Table 9, Table 10 and Table 11, it is observed that although every model performance is good, and ANFIS dominates the rest of the metamodels.

## 5. Conclusions

Scatter plots, probability distribution plots, and statistical parameters presented and discussed above show that there is a negligible deviation of GPR, MARS, and ANFIS model from MCS-FEM, which indicates the high precision of these metamodels. However, the deviation of PSO-ANN predicted mode shape frequencies from MCS-FEM is notable and shows that its precision concerning the rest of the metamodel is less. Notably, the present metamodels are trained with different design points and corresponding mode shape frequency evaluated with MCS-FEM. Further, these trained metamodels evaluate 3000 results for each mode shape within seconds to describe the scatter plots, probability distribution plot, and statistics of the four-mode shape frequencies of a hybrid angle ply laminated composite plate. Though the same number of frequency, i.e., 3000, is evaluated with MCS-FEM for describing the scatter plots, the probability distribution plot of mode shape frequencies of a hybrid laminated composite plate takes more than 24 h on a work station (a powerful computation tool). For the metamodels, the number of FEM simulations required is much less compared to MCS-FEM, as with limited design points, metamodels are trained and 3000 results are evaluated within seconds. Henceforth, when the analysis is performed with metamodels, the computational time, cost, and effort are considerably reduced concerning MCS-FEM. This offers an effective and inexpensive way of simulating the stochasticity in mode shape frequencies. The best possible number of finite element simulations requisite to train the metamodels are determined by evaluating the RMSE value. In the present analysis, all the layer-wise combined cases of stochasticity are studied as described in the random input representation section.

- The novelty of the article is a probabilistic description of four mode shapes of hybrid angle ply laminated composite plates with MCS-FEM and efficient metamodels GPR, MARS, PSO-ANN, and ANFIS and showing the advantage of metamodels over the FE model, as in earlier published articles, no or very limited work is found on mode-shape analysis using soft computation metamodels.
- Mode-frequency always increases from the 1st to 4th mode and remains the same in very limited cases. The first, second, third, and fourth non-dimensional mode-frequency parameter monotonically increases, and there is very little difference between 3rd and 4th mode-frequencies. This monotonous increase is due to an increase in the degree of orthotropy.
- The regression plot and probability distributions plots of various mode shapes are analyzed by taking the combined variation of material property.
- The present work explains the effect of stochasticity on various mode shapes of a hybrid angle ply laminated composite plate by employing the MCS-FEM and metamodels.
- Various metamodels are formed along with MCS-FEM to characterize the variation of mode shape frequencies produced due to uncertainty in material properties, where it is witnessed that the number of finite element simulations is remarkably shrunk compared to MCS-FEM without compromising the precision of the result, thereby reducing the computational cost, time, and effort.
- The data shown in the study show that uncertainty in material properties of the hybrid angle ply laminated composite plate has a substantial influence on the dynamics of the composite structure and its various mode shapes. Hence, it is significant to count the uncertainty in the analyses, design, and control of the hybrid angle ply laminated composite plate.
- The anticipated GPR, MARS, PSO-ANN, and ANFIS metamodel-based stochastic analysis may be stretched further to analyze other stochastic systems in the forthcoming research.

## Figures and Tables

**Table 1:** Details of the parameters of the ANFIS model.

Parameter	Value
Fuzzy structure	Sugeno-type
Initial FIS for training	Genfis3
Maximum Iterations Number	1000
Number of Fuzzy rules	15
Input MF type	Gaussian
Output MF type	Linear
Initial Step size	0.01
Step Size Decrease Rate	0.9
Step Size Increase Rate	1.1

**Table 2:** (**a**) Convergence and comparison of non-dimensional frequency parameter of simply supported cross-ply laminate; (**b**) comparison study of the fundamental frequency of simply supported cross-ply square laminate with HSDT C^1^ finite element model; (**c**) comparison study of the fundamental frequency of simply supported cross-ply square laminate with HSDT *C*^0^ finite element model.

(**a**)						
***h/a***	**Lamination**	**References**	**Mode**			
			**1**	**2**	**3**	**4**
0.01	0°/90°/0°	8 × 8	18.866	27.153	47.920	71.140
		12 × 12	18.848	26.969	46.723	70.757
		16 × 16	18.848	26.939	46.493	70.709
		18 × 18	18.848	26.922	46.358	70.676
		20 × 20 (Present)	18.848	26.922	46. 358	70.675
		[48]	18.838			
		[49]	18.733			
0.1		8 × 8	15.108	24.295	24.334	26.462
		12 × 12	15.107	24.317	24.334	26.445
		16 × 16	15.107	24.325	24.334	26.442
		18 × 18	15.107	24.328	24.334	26.442
		20 × 20 (Present)	15.107	24.328	24.334	26.441
		[48]	15.214			
		[49]	15.145			
(**b**)						
**Reference**			***a/h***			
			**5**		**10**	**50**
HSDT C^1^ FE [50]			9.087		10.569	11.276
Present			9.085		10.571	11.314
(**c**)						
**Reference**			***a/h***			
			**5**		**10**	**50**
HSDT *C*^0^ FE [51]			8.660		10.382	11.287
Present			9.085		10.571	11.314

**Table 3:** Relative material property of two different materials used in hybrid angle ply laminated composite plate.

Sl. No.	Material 1 Relative Property	Material 2 Relative Property	Variation Provided in the Material Property (%)
1	*E*_1_ = 25	*E*_1_ = 40	±10
2	*E*_2_ = 1	*E*_2_ = 1	±10
3	*υ*_12_ = 0.25	*υ*_12_ = 0.25	±10
4	*υ*_21_ = 0.01	*υ*_21_ = 0.00625	±10
5	*G*_12_ = 0.5	*G*_12_ = 0.6	±10
6	*G*_13_ = 0.5	*G*_13_ = 0.6	±10
7	*G*_23_ = 0.2	*G*_23_ = 0.5	±10
8	*ρ* (density) = 1.0	*ρ* (density) = 1.0	0

**Table 4:** Statistical description of target and predicted dataset for 1st mode shape.

Combined Variation of Material Property	Frequency for 1st Mode Shape					
	Value	MCS-FEM	GPR	MARS	PSO-ANN	ANFIS
*S*(ϖ)	Max	1.5609	1.5716	1.5645	1.5474	1.5621
	Min	1.1411	1.1487	1.1461	1.1885	1.1480
	Mean	1.3398	1.3398	1.3398	1.3399	1.3398
	SD	0.0631	0.0631	0.0631	0.0628	0.0631

**Table 5:** Statistical description of target and predicted dataset for 2nd mode shape.

Combined Variation of Material Property	Frequency for 2nd Mode Shape					
	Value	MCS-FEM	GPR	MARS	PSO-ANN	ANFIS
*S*(ϖ)	Max	3.0034	2.9982	3.0111	2.9171	3.0062
	Min	2.1724	2.1789	2.1777	2.2770	2.1740
	Mean	2.5667	2.5668	2.5667	2.5673	2.5667
	SD	0.1252	0.1251	0.1252	0.1238	0.1252

**Table 6:** Statistical description of target and predicted dataset for 3rd mode shape.

Combined Variation of Material Property	Frequency for 3rd Mode Shape					
	Value	MCS-FEM	GPR	MARS	PSO-ANN	ANFIS
*S*(ϖ)	Max	4.6979	4.6613	4.7064	4.5615	4.7015
	Min	3.3932	3.3937	3.4045	3.5581	3.3962
	Mean	4.0128	4.0127	4.0127	4.0138	4.0128
	SD	0.1964	0.1963	0.1964	0.1942	0.1964

**Table 7:** Statistical description of target and predicted dataset for 4th mode shape.

Combined Variation of Material Property	Frequency for 4th Mode Shape					
	Value	MCS-FEM	GPR	MARS	PSO-ANN	ANFIS
*S*(ϖ)	Max	4.7304	4.7108	4.7425	4.5935	4.7339
	Min	3.4158	3.4181	3.4245	3.5818	3.4192
	Mean	4.0403	4.0403	4.0403	4.0413	4.0404
	SD	0.1980	0.1979	0.1980	0.1958	0.1980

**Table 8:** Statistical parameters for the 1st mode shape results predicted with various metamodels.

Parameter	GPR	MARS	PSO-ANN	ANFIS
RMSE	0.0011	0.0011	0.0047	0.0009
NS	0.9999	0.9999	0.9999	0.9999
RSR	0.0009	0.0009	0.0039	0.0008
VAF	99.9681	99.9717	99.4386	99.9792
*R*	0.9998	0.9999	0.9972	0.9999
*R* ^2^	0.9999	0.9997	0.9944	0.9998
Adj.*R*^2^	0.9997	0.9997	0.9944	0.9998
PI	1.9982	1.9984	1.9840	1.9987

**Table 9:** Statistical parameters for the 2nd mode shape results predicted with various metamodels.

Parameter	GPR	MARS	PSO-ANN	ANFIS
RMSE	0.0006	0.0007	0.0099	0.0005
NS	1	1	0.9999	1
RSR	0.0002	0.0003	0.0041	0.0002
VAF	99.9979	99.9971	99.3688	99.9984
*R*	0.9999	0.9999	0.9969	0.9999
*R* ^2^	0.9999	0.9999	0.9938	0.9999
Adj.*R*^2^	0.9999	0.9999	0.9937	0.9999
PI	1.9994	1.9993	1.9774	1.9995

**Table 10:** Statistical parameters for the 3rd mode shape results predicted with various metamodels.

Parameter	GPR	MARS	PSO-ANN	ANFIS
RMSE	0.0009	0.0007	0.01560	0.0004
NS	1	1	0.9999	1
RSR	0.0002	0.0002	0.0040	0.0001
VAF	99.9977	99.9985	99.3712	99.9996
*R*	0.9999	0.9999	0.9968	0.9999
*R* ^2^	0.9999	0.9999	0.9938	0.9999
Adj.*R*^2^	0.9999	0.9999	0.9937	0.9999
PI	1.9990	1.9992	1.9719	1.9996

**Table 11:** Statistical parameters for the 4th mode shape results predicted with various metamodels.

Parameter	GPR	MARS	PSO-ANN	ANFIS
RMSE	0.0006	0.0008	0.0157	0.0004
NS	1	1	0.9999	1
RSR	0.0002	0.0002	0.0040	0.0001
VAF	99.9989	99.9983	99.3707	99.9997
*R*	0.9999	0.9999	0.9968	0.9999
*R* ^2^	0.9999	0.9999	0.9937	0.9999
Adj.*R*^2^	0.9999	0.9999	0.9937	0.9999
PI	1.9994	1.9991	1.9717	1.9996

## Data Availability

Data and methods used in the research has been already presented with sufficient detail in the paper so that other researchers can replicate the work.

## Nomenclature

*a*, *b*, *h*	Dimension of the plate along x, y, and z axes, respectively	{*ε*}, [*B*_*i*_]	Global displacements and differential operator matrix
*μ*, *μ_o_*, *θ*_*y*_	Displacement, midplane displacement, and rotation along the x-axis respectively	[m], |*j*|	Inertia matrix and determinant of the Jacobian matrix
*v*, *v*_*o*_, *θ*_*x*_	Displacement, midplane displacement and rotation along the y-axis, respectively	*k*, *m*	Element stiffness matrices
*w*, *w*_*o*_	Displacement, midplane displacement along the z-axis	(*f*(*x*)), *m*(*x*) *θ*, *k* (*x*, *x*′)	Gaussian process function (GP), mean of GP, Hyperparameter of GP and Covariance of GPR respectively
*φ*_*x*_, *χ*_*x*_, *φ*_*y*_, *χ*_*y*_	Higher-order terms of Taylor series expansion	*β_o_*, *β_n_*, *BF*	Intercept in MARS, coefficient corresponding to given specific function and Basis function, respectively
*ε**x**x*, *ε**y**y*, *γ**x**y* *γ**x**z*, *γ**y**z*	Strain displacement terms	*x_i_*, *U_i_*	Position vector and velocity vector
*E*_1_, *E*_2_, *v*_1_, *v*_2_	In-plane Modulus of elasticity and Poisson’s ratio	*G*_12_, *G*_13_, *G*_23_	Modulus of rigidity (in-plane and out of plane)
{*d*}, *N**i*	Unknown nodal vector on middle surface of the element and interpolating shape function respectively	$P_{best,i}^{k},\;G_{best,i}^{k}$	Local best fitness function and global best fitness function respectively
*T*_kl_	Target value	*P*_kl_	Predicted value
*A*_1_, *A*_2_, *B*_1_, *B*_2_	Nonlinear parameter and membership function for input variables	*a*_1_, *a*_2_, *b*_1_, *b*_2_, *r*_1_, and *r*_2_	Linear and output’s function parameters
*MSE_train_*	Mean squared error of the model	*p_eff_*	Effective parameters
*ρ*, *E*2	The density of material and Modulus of elasticity in a transverse direction.	*E*	The fitness value of PSO-ANN model
*λ*, *w*	Non-dimensional frequency parameter and dimensional frequency parameter		
